# The Impact of Different Powdered Mineral Materials on Selected Properties of Aerobic Granular Sludge

**DOI:** 10.3390/molecules25020386

**Published:** 2020-01-17

**Authors:** Joanna Czarnota, Adam Masłoń, Monika Zdeb, Grzegorz Łagód

**Affiliations:** 1 Department of Environmental Engineering and Chemistry, Rzeszow University of Technology, 6 Powstańców Warszawy Av, 35-959 Rzeszów, Poland; amaslon@prz.edu.pl; 2 Department of Water Purification and Protection, Rzeszow University of Technology, 6 Powstańców Warszawy Av, 35-959 Rzeszów, Poland; mzdeb@prz.edu.pl; 3 Faculty of Environmental Engineering, Lublin University of Technology, Nadbystrzycka 40B, 20-618 Lublin, Poland

**Keywords:** aerobic granular sludge, new wastewater treatment technology, physical and chemical properties of granules, biological properties, powdered mineral materials

## Abstract

This study aimed to evaluate and compare the physical, chemical and biological properties of aerobic granular sludge from reactors with the addition of different powdered mineral materials. These properties have a significant impact on the efficiency of systems in which the biomass in granular form is used. Four identical granular sequencing batch reactors (GSBRs) were adopted for the research performed on a laboratory scale (R1—control reactor; R2, R3 and R4—with materials, PK, PG and PL respectively). The results indicate that the addition of powdered mineral materials improved the properties of biomass in reactors. The SVI_5_/SVI_30_ ratio values were significantly lower in the reactors with added materials (approx. 1.3 ± 0.3). The mean values of the sludge volume index at 30 min were the lowest in the R2 (39.8 ± 8.6 mL/g) and R4 (32.8 ± 10.7 mL/g) reactors. The settling velocity of biomass was the highest in the R2 reactor (15.4 ± 6.1 m/h). In the early days of the study, the highest extracellular polymeric substances (EPS) content was found in the biomass from the reactors to which the materials with higher Ca and Mg content were added (380.18–598.30 mg/g MLVSS). The rate of specific oxygen uptake (SOUR) by biomass indicated an insufficient biomass content in the R1 reactor—to 7.85 mg O_2_/(g MLVSS∙h)—while in the reactors with materials, the SOUR values were at the higher levels.

## 1. Introduction

In recent years, actions have been taken to improve and intensify the technology of activated sludge [1,2,3]. The previous actions in this area led to the development of a method using such sludge in the form of granules. A granulation process of this kind can occur under conditions that are both anaerobic (UASB—*Upflow Anaerobic Sludge Blanket*) and aerobic (AGS—*Aerobic Granular Sludge*). The use of SBR (*Sequencing Batch Reactor*) technology allows for such changes in the structure of activated sludge biomass as can transform the flocs of sludge into aerobic granules. While the biomass in the form of such granules has many characteristic parameters, Gao et al. [4] sought to classify these in a straightforward way as physical, chemical and biological. These properties have a significant impact on the efficiency of systems in which the biomass in the form of granules is used.

The group of physical properties of aerobic biomass includes the morphology and size of granules, spatial structure, sedimentation properties, porosity, specific gravity, density of granules, water content and physical strength [4,5,6,7,8]. The physical parameters of granular biomass are important due to the stability of the granules obtained, but also given the nature of the biological processes by which wastewater is treated [9]. Some of these parameters are taken into account as aerobic granular sludge is defined. According to the literature, granules are aggregates of grain size in the 200 µm–16 mm range, which are formed without a biomass carrier needing to be deployed [10]. Furthermore, the cultured granules are considered mature when 90% of them have a diameter over 200 μm [11], or when the difference between SVI_5_ (sludge volume index after 5 min) and SVI_30_ (sludge volume index after 30 min) for the same aerobic granular sludge is less than 10%, and the granules are of a clearly defined shape [12].

The chemical properties of aerobic granular sludge are evaluated in terms of the hydrophobicity of the cell surface and the content of extracellular polymeric substances (EPS) [4,13]. The latter substances are assigned an important role in the biogranulation process [14], as components of aerobic granules do much to shape the chemical structure and physical composition of AGS [15]. Tu et al. [16] regard EPS as a factor of decisive relevance to the mechanical properties of granules. The content of EPS in granular biomass is seen to be affected significantly by the presence of cations that are divalent (Ca^2+^, Mg^2+^, Fe^2+^) or trivalent (Fe^3+^) [9,14]. In turn, cell-surface hydrophobicity initiates cell aggregation, as an important stage in the biogranulation process [17].

The biological properties of AGS reflect the microbiological composition [4]. It should be recalled that aerobic granules are classified as a consortium of self-immobilised microorganisms, consisting mainly of aerobic and facultative bacteria, and hence being active in nature [10]. However, in the process of biogranulation, an important role is assigned to filamentous bacteria, which form the framework in the initial stage at which aerobic aggregates form [18]. Where organic loading rate (OLR) values are low, leaving the number of filamentous bacteria proportionally higher, granules tend to disintegrate [19], or else are present in a specific form of biomass assuming a black colour [18].

Recently, research has been conducted to intensify the process of aerobic biogranulation and enhance the efficiency of AGS technology. One of the new research directions in this area is the application of powdered material with a particle size of less than 300 μm at the start-up stage of a granular sequencing batch reactor [20]. The literature review shows that organic and mineral powdered substances have been used for this purpose. A number of materials from the first group were used, including powdered activated carbon (PAC) [21,22], dry sewage sludge micropowder [23], granular activated carbon (GAC) [24,25,26,27], and biochar [28]. The mineral materials used to date were zeolite [29], bivalve shell carrier [30,31], yellow earth [32], ceramsite [8,20] and basalt [17]. According the literature [20,23,27,31,32], adding such materials to a reactor results in an improvement of the sedimentation properties of the obtained aerobic granules, as well as allowing the acceleration of microorganism aggregation and the formation of mature granules in a short time. The detailed characteristics of the materials used to date in AGS technology and comparison of the granule properties in the systems with and without powdered substance were presented in the article Czarnota et al. [20]. The studies carried out to date have shown that especially organic powdered materials were an excellent biomass microcarrier, which determines the acceleration of the biogranulation process [21,23,25,27]. In turn, both groups of powdered materials represent a ballast of sludge flocs, especially in the few initial cycles after start-up of a reactor, which prevents the excessive washing-up of biomass from the reactor and, as a result, allows obtaining aerobic granules [20,23,24,29,31]. Thanh et al. [30] stated that powdered materials, applied in AGS technology, should: be heavy enough, have a spherical shape, sediment quickly, be steadily suspended with biomass, and have a large specific surface area. Additionally, Czarnota et al. [20] stated that the divalent cations contained in powdered mineral materials can participate in the formation of the matrix of aerobic granules. Furthermore, the efficiency of organic compounds, nitrogen and phosphorus removal from wastewater in reactor supported by powdered materials was higher than in control reactor [20,22,25,26,29]. Moreover, Li et al. [24] reported that compared to the aerobic granules from the reactor without GAC, these GAC enhanced granules withstood a lower organic loading rate.

Thus, the work detailed here sought to evaluate selected physical, chemical and biological properties of aerobic granular sludge grown in reactors supplied with different powdered mineral materials. The evaluation in question thus pertained to the physicochemical properties of the materials used, e.g., their specific surface area, grain size, chemical composition, and the leaching of elements important to granule formation.

## 2. Results and Discussion

### 2.1. Selected Physical Properties of Biomass

During the period of system adaptation, the volume of reactors was dominated by flocs of activated sludge. It is worth mentioning that in microscopic images, the flocs serving as inoculum were assessed as small (52.3 ± 10.7 µm), not very compact and irregularly shaped. The inoculum was also found to have a small proportion of flocs of compact structure and irregular and jagged edges. The parameters such as mixed liquor suspended solids (MLSS) and sludge volume index after 30 min (SVI_30_) for inoculum were of about 6.40 g MLSS/L and 135.2 mL/g, respectively. In the previous studies on the aerobic biogranulation process supported by powdered materials as inoculum, an activated sludge was usually used. However, it should be emphasized that the parameters of activated sludges used as inoculum were different. For example, in article of Zhou et al. [25], the seed sludge had a loose morphology with a mean diameter of 62 ± 2.4 µm and SVI_30_ was approximately 125 ± 17 mL/g. In turn, Li et al. [23] used activated sludge with SVI_30_ of 167 mL/g or bulking sludge with SVI_30_ of 225 mL/g as inoculum. The authors showed that in the reactor with bulking sludge as seed sludge and by adding micropowder, filamentous bacteria were controlled and granules were formed.

**Figure 1:**
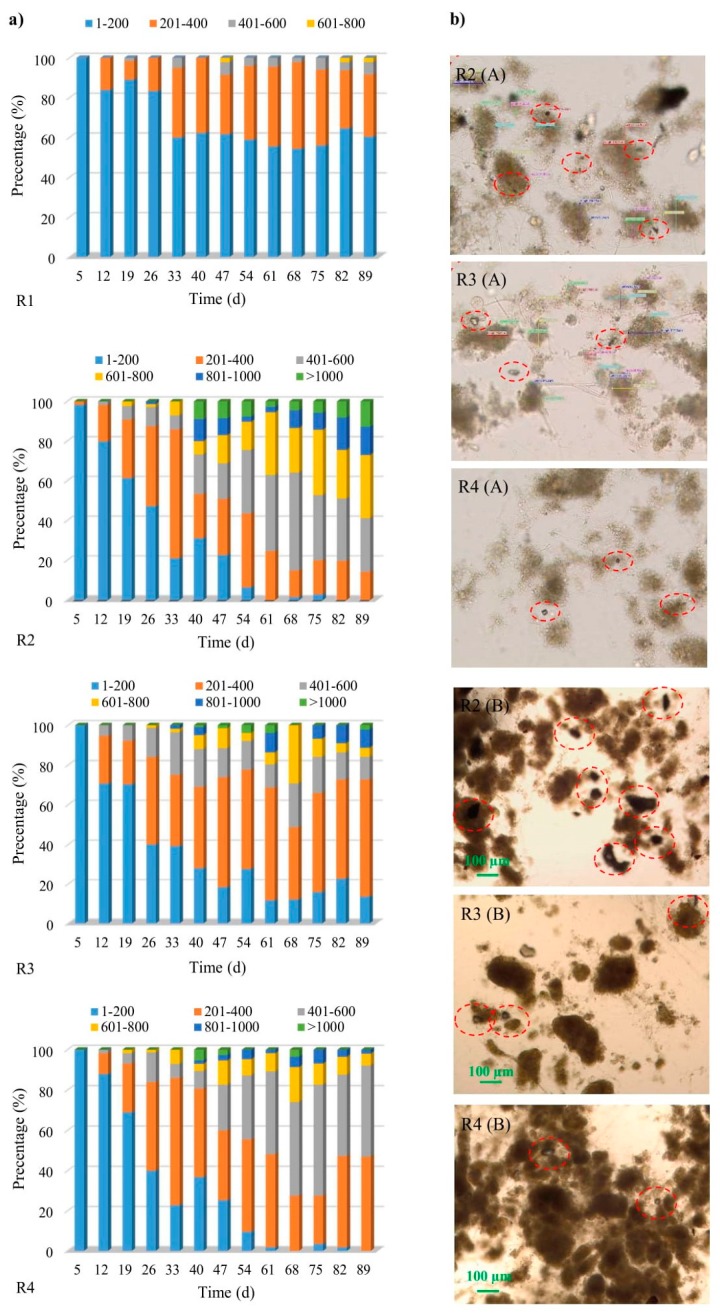
The biogranulation process including the percentage of granules of a given range of diameters (**a**), and the powdered mineral materials in the structures of flocs and granules: A—5th day of research, B—19th day of research (**b**).

The first granules were observed as early as on day 5 of the study, in the reactor with powdered ceramsite (R2). By approximately the 12th day, granules had appeared in all the reactors and accounted for about 16% (R1), 20% (R2), 29% (R3) or 12% (R4), respectively. The further process of biogranulation was different from one reactor to another (Figure 1a). The time of appearance of the first granules in own research is comparable with some of the literature data. As described by Minh [17], who used basalt in his research, the first granules in the reactor were present on day 7.

More intensive granule formation was observed, for example, in the reactors with powdered granite (R3) and powdered ceramsite (R2). In the control reactor (R1), from the 33rd day of the research onwards, flocs of activated sludge accounted for about 60%, while the proportion of granules of about size 0.40–0.60 mm was only 6%. In R2 and R4, after day 54 of the study, particles smaller than 0.2 mm accounted for less than 10% of the total, indicating complete biogranulation in line with the definition. In addition, in these two reactors, the difference between SVI_5_ and SVI_30_ was in practice below 10% from the 40th day, with this also offering confirmation that aerobic granular sludge had been obtained. Stable granules were obtained in the reactor to which powdered ceramsite had been added, as no significant increase in the percentage occurrence of smaller granules was observed subsequently. An opposite situation was observed in the reactors with limestone and granite, where percentage increase of smaller granules occurred after 75th day and 68th day, respectively. Moreover, in the R2 reactor after day 40, a stabilization of the EPS values was observed (this aspect is discussed in detail in Section 2.2), which indicates the maturation process of granules [4]. In the research of Zhou et al. [25], who added 0.2 mm GAC to the reactor, the biogranulation process occurred on the 31st day. On the other hand, in the reactor without GAC and in the reactor with 0.6 mm GAC, no significant biogranulation was observed. Such observations allow to conclude that the grain size of organic powdered material translates into the biogranulation process. Li et al. [22] showed that the addition of GAC prominently shortened the formation of aerobic granules (from 6 weeks to less than 3 weeks). However, the same authors showed that the addition of PAC did not considerably accelerate biogranulation. Wei et al. [29] reported that the strategy of adding zeolite carrier shortened the formation time of aerobic granular and reduced the risk of filamentous bulking. The authors stated that the process of forming mature granules in a reactor without zeolite and in a reactor with the addition of zeolite lasted for 90 days. However, the average diameter of the obtained granules was 1.5 mm and 2.5 mm, respectively. While comparing the literature data, it should be remembered that the operating conditions of the reactors in individual articles were different. It should be borne in mind that certain parameters (especially OLR) determine the biogranulation process.

From the 19th day of the study onwards, granules were of a much larger average size in the reactors supplied with powdered mineral materials. Equally, from day 33 onwards, the average values were significantly higher in the reactor treated with powdered ceramsite. After day 68, decreases in the average sizes of the aggregates were visible in the reactors supplied with both granite and limestone.

A more stable growth of the granules was observed in R2, in which larger aggregates were present, owing to the application of that particular material. Microscopic observations indicate the uniform suspension of powdered ceramsite with biomass, as evidenced by the presence of visible particles of this material within the structures of activated-sludge flocs and granules. The same true for granite and limestone, only to a lesser extent. Out of the materials applied, the powdered ceramsite gives rise to the greatest specific surface area. The materials used in the tests had an incomparably low specific surface area (1.760–5.183 m^2^/g) in relation to the powdered organic materials, for example, activated carbon (500–1000 m^2^/g), used in AGS technology [20]. Moreover, it was possible to observe the formation of granules containing microparticles of powdered ceramsite (Figure 1b). A positive effect of powdered mineral materials particles on the sedimentation properties of sludge in reactors was also noted (Table 1).

The SVI_5_/SVI_30_ ratio values were significantly lower in the reactors supplied with the powdered mineral materials. After 54 days, the values for this parameter were higher in the reactor with granite, a circumstance influenced by a significant decrease in MLSS in this reactor. The average SVI_30_ value in the reactor with powdered ceramsite (PK) coincides with that noted by Wei et al. [21], who used powdery activated carbon (38.0 mL/g). The SVI_30_ in the reactor with powdered granite (PG) is comparable with the value presented by Li et al. [23], who used dry sewage sludge micropowder as a powdered material (51.0 mL/g). In the reactor with powdered limestone (PL), the sludge volume index coincides with that from Wei et al. [29], who applied zeolite to a reactor (34.9 mL/g). In the reactors with mineral materials, a higher settling velocity was observed (highest of all in R2, slightly lower in R4) (Table 1). However, it should be borne in mind that the application of powdered mineral materials, in general, increased the settling velocity rates in reactors, while the course of the studies also allowed for observation of the biomass biogranulation process. Aggregates with much larger diameters were found primarily in the reactors with powdered ceramsite and limestone. Our own research and previous research clearly showed that powdered materials, whether organic or mineral, improved the sedimentation properties of biomass in reactors. The improvement of these properties translated into a reduction of the biomass washing-up from the system at the initial stage of research, which often determined the further biogranulation process.

The density of granules also changed in the course of studies. In the reactor with PK, this parameter reached its highest value, oscillating between 26.12 and 37.26 g MLVSS/L_granules_. A slow increase in this parameter was also to be observed in subsequent days. High density values were also recorded in the reactor with PL (from 21.45 to 30.47 g MLVSS/L_granules_), while in this case, after 54 days of testing, the values for this parameter decreased. However, in the reactor with PG, the concentration of sedimentation biomass manifested a downward trend with the duration of the research (from 39.60 to 16.69 g MLVSS/L_granules_). The lowest biomass density was observed in the R1 reactor (about 15.2 g MLVSS/L_granules_). The results obtained are comparable with those to be found in the literature. Minh [17] used basalt and an OLR equal to 3.0 g COD/(L∙d) in a study, and the obtained sedimentation biomass concentration was 28.7 g MLVSS/L_granules_ in a SBAR (*Sequencing Batch Airlift Reactor*), and 30.4 g MLVSS/L_granules_ in a SBBR (*Sequencing Batch Bubble Reactor*). The author showed that with increasing organic loading, the density of granules is found to increase. At an OLR of 20.0 g COD/(L∙d), the respective parameters were 47.8 and 53.7 g MLVSS/L_granules_ respectively. Thanh et al. [30], using a bivalve shell carrier and an OLR of 2.50 g COD/(L∙d), obtained a granule density of 25.0 g MLVSS/L_granules_. An increase in the load to 30.0 g COD/(L∙d) ensured an increase in density to 49.2 g MLVSS/L_granules_. In turn, in the research by Arrojo [33], the density of biomass was in the range 10.0–30.0 g MLVSS/L_granules_, while a rapid increase occurred during the period in which the nitrifying bacteria were recorded as dominant in the system. In the authors’ own research in the R1, R3 and R4 reactors, the density of granules was found to depend on the MLVSS concentration. On the other hand, in the R2 reactor, the MLVSS values were at a comparable level from the 68th day of the research onwards, while the concentration of sedimenting biomass increased.

The biomass in all the reactors differed quite markedly with regards to total granule volume, the approximate number of granules and the specific surface area of the granules in the reactor (Table 2).

In a study by Dezotti et al. [35], biomass achieving a density of 48.0 g MLVSS/L_granules_ and a concentration of 4.0 g MLVSS/L was characterised by a specific surface area equal to 785 m^2^/m^3^. Such values for individual parameters ensured the presence of both the nitrifying and denitrifying bacteria in the system. However, when the biomass density was 15.0 g MLVSS/L_granules_, at a concentration of 0.7 g MLVSS/L, the specific surface area of the granules was 1700 m^2^/m^3^, and no denitrification process was to be observed in the reactor. On the other hand, Beun et al. [34] reported that a specific surface area of biomass of about 2300 m^2^/m^3^ coincided with respective figures for the density and concentration of granules equal to 60.0 g MLVSS/L_granules_ and 4.0 g MLVSS/L, with autotrophic biomass being dominant within the reactor.

The spatial form of aerobic granule sludge was evaluated using industrial computed tomography. The granules cultured in the control reactor were characterised by a fluffy surface with filamentous bacteria, which dominated in the granules and contributed to a loosening of structure. On the other hand, the granules from the reactors supported by powdered mineral materials were characterised by a non-fibrous and smoother surface on which corrugations were visible, most probably as a result of the porosity of these aggregates being greater. The application of the materials enabled to obtain a more compact structure of aerobic granular sludge. The addition of powdered materials also served to limit the development of filamentous microorganisms on the surfaces of granules. These bacteria provide the framework of the aggregates obtained. Similar observations were presented by Li et al. [23].

### 2.2. Selected Chemical Properties of Biomass

**Figure 2:**
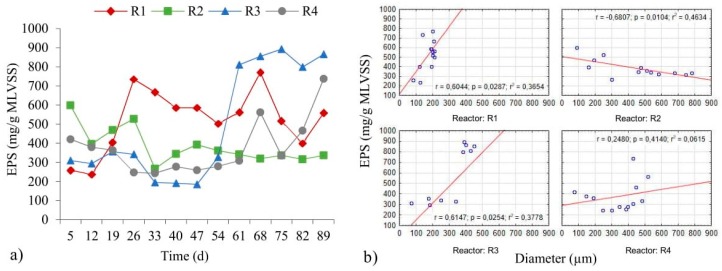
Variations of EPS in successive days (**a**), as well as the relationship between amount of EPS and diameters of the granules (**b**).

The average values for EPS in the biomass obtained were: 522.0 ± 163.3 mg/g MLVSS (R1); 385.4 ± 93.3 mg/g MLVSS (R2); 494.2 ± 294.3 mg/g MLVSS (R3) and 375.6 ± 143.8 mg/g MLVSS (R4). Objectivity should be maintained when the obtained values are compared with the data in the literature on account of the fact that the differences in EPS values, for example, may result from the adapted methodology [36]. In the article by Rusanowska et al. [37] (COD in inflow was 700.0 mg O_2_/L), the average EPS values in aerobic granular sludge—as determined by sonication and an ion-exchange resins method [37]—were 581.4 and 46.9 mg/g MLSS respectively. The authors reported that the values for proteins and polysaccharides in total EPS content, as obtained using the first method, were 1.9 and 213.2 mg/g MLSS respectively. Caudan et al. [36] showed that the protein content reported tends to be higher when gauged using the sonication method. The results of the study presented here confirm the changes in the amounts of extracellular polymeric substances in successive days of the experiment (Figure 2a).

In the early days of the study, the highest EPS content was found in the biomass of the reactors to which powdered ceramsite and powdered limestone had been added. In comparison with powdered granite, these materials have greater amounts of Ca and Mg in their composition. It can be concluded that—in the first few days of the reactor operation—the materials were an additional source of cations stimulating biogranulation. Kończak [14] stated that multivalent cations influence the components of extracellular polymeric substances. Between the 26th and 54th days, the greatest amounts of EPS were found in the biomass from the R1 reactor, which most likely resulted from a much higher proportion of activated sludge flocs in biomass. Sheng et al. [38] showed that smaller and loose-biomass aggregates differ from larger and denser aggregates in being characterised by higher EPS production. The increase in the amount of EPS in the R3 and R4 reactors after 60 days resulted from the washing out of biomass from reactors, as caused by the breakup of granules. In the R1, R3 and R4 reactors, the amount of extracellular polymeric substances increased with granule development (Figure 2b), with this pointing to the absence of mature and stable granules. Deng et al. [39] reported that the EPS content is determined once stable granules have formed. The correlation between these parameters for R1 and R3 was positive and statistically significant (*p* < α, *p* = 0.0287 for R1, *p* = 0.0254 for R3). However, in the R2 reactor, a high inverse correlation was observed (Figure 2b), as well as the amount of EPS after 47 days at a similar level (Figure 2a), based on Kończak [14] and Campo et al. [40]’s reports, which confirms the achievement of stable aggregates. Moreover, given that after day 47, an increase the size of granules was observed in the R2 reactor which were compact and their structure was stable, it can be stated that after this day, the maturation process of granules ensued. Campo et al. [40] showed that the maturation process includes an increase of granules’ average diameter, stabilized of EPS values and appeared of granules which are compact, rounded and with a regular shape.

The dependent relationship noted between the EPS value and the diameter of granules in the R2 reactor did achieve statistical significance (*p* < α, *p* = 0.0104). The observations from the authors’ own research are comparable with those from Sheng et al. [38], who recorded lower values of EPS in successive days of an experiment in a reactor with larger granules. In turn, in the study by Wei et al. [21], the EPS content in the control reactor and that with powdered activated carbon were at comparable levels, while in the work presented here, it was possible to observe significant differences between the reactors. The different EPS content in the reactors could be due to the presence of powdered materials characterised by a different chemical composition as well as the progressing biogranulation process.

**Figure 3:**
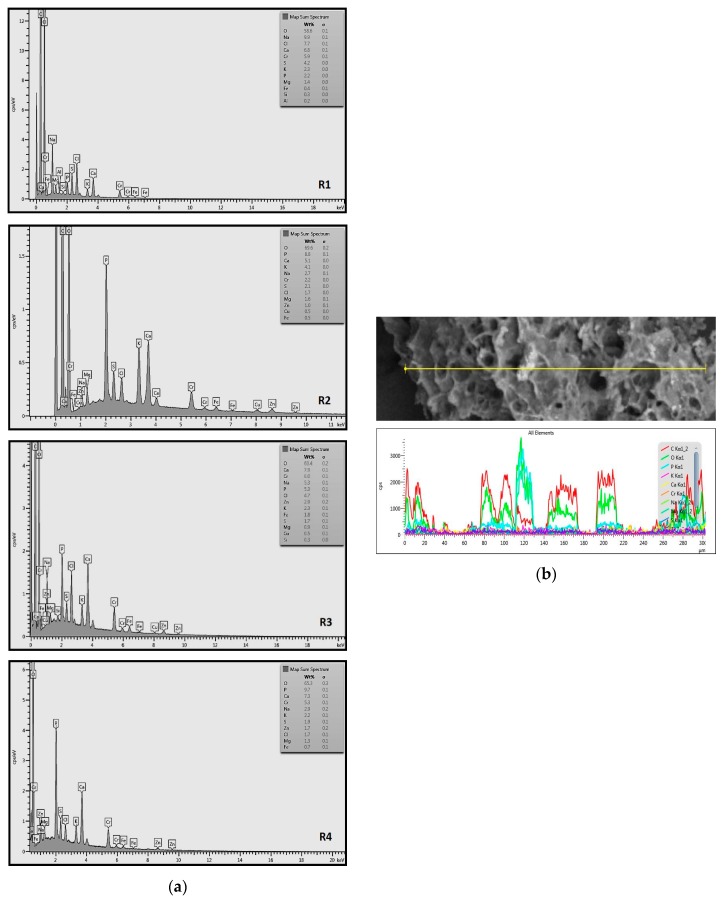
EDS spectra of the elemental composition Aerobic Granular Sludge (AGS) for the measurement area according to Figure 4b (**a**) and the elemental composition (qualitative) of the fragment of the granule from reactor R2 (**b**).

Efforts to identify the elements included in the biomass showed that in the structure of granules, carbon and oxygen were accompanied by calcium, phosphorus, magnesium, sodium, iron, potassium and sulphur. In the granules from the reactors supported by powdered materials, zinc and copper were additionally present. The analysis of the approximate percentage content of elements in the sample (wt%) showed, inter alia, a higher proportion of oxygen and phosphorus in the granules grown in the reactors to which materials had been added. The lowest content of calcium was found in the granules from the R2 reactor and the highest from the R3 reactor. The percentage occurrence by weight of magnesium and iron was comparable in biomass from the R1, R2 and R4 reactors. However, in the granules from the R3 reactor, the Mg content was only around half as high, while the amount of Fe was more than double (Figure 3a).

In the studies carried out by Mañas et al. [41], the percentage by weight of individual elements in the aerobic granular sludge grown on wastewater with a higher concentration of Ca and PO_4_^3−^ (respectively about 1 mmol Ca/L and 30 mg P/L), was as follows: 12.87% P; 56.33% O_2_; 0.89% Mg; 28.75% Ca; 0.95% Na and 0.2% S. The authors reported that the main mineral in the granules rich in calcium and phosphorus was hydroxyapatite. The value of the Ca/P ratio in the granules obtained was 1.63 and corresponded to Ca/P for hydroxyapatite. The results from the SEM-EDS analysis suggest that calcium phosphate compounds such as amorphous calcium phosphate (Ca/P = 1.5) were present in the granules grown in the R3 reactor. The Ca/P ratio was calculated after Mañas et al. [41]. In the reactors with PK and PL, the Ca/P ratio was below 1. Cydzik-Kwiatkowska et al. [42] reported that the dose of the external carbon source is a decisive factor affecting the elemental composition of biomass. The granules cultured at a dose of 540 mg COD/(L∙cycle) had a higher percentage by weight of C, S and N, which indicated the presence of greater amounts of organic compounds in the structure of the granular sludge. At a lower dose of the external carbon source in biomass, it was possible to note higher contents of Mg, Ca and P. The granules from the biogranulation process supported by dry sewage sludge micropowder were of the following approximate composition: 16% Ca; 15% P; 3% Mg; 5% Fe, K and Si [23]. In the authors’ own research, it was noted that although the reactors operated at the same organic loading rate (2.55 g COD/L∙d), the F/M (food-to-microorganism) values might still be diverse. The F/M value was highest in the control reactor and amounted to 1.796 g COD/(g MLVSS∙d). In reactors with powdered materials, the relevant values were lower, at 0.637 (R2); 1.142 (R3) and 0.763 g COD/(g MLVSS∙d) (R4). In the R1 and R3 reactors, operating at higher F/M values, large Ca, Mg and P “crystals” were recorded on the elemental (qualitative) maps devised (Table 3). The precipitation of the mineral compounds in the granules in the R3 reactor might have limited the diffusion of oxygen, leading to the breakup of the granules. Toh et al. [43] suggested that it was the limiting of diffusion by oxygen and nutrients inside granules that contributed to their disintegration. In order to assess elemental composition more accurately, measurements were conducted at several locations in the measurement area. For the granules from the R2 and R3 reactors, a certain repeatability of the weight fraction of individual elements was to be noted (Figure 3b). On the other hand, large differences in the percentages of individual elements (other than oxygen) were to be noted in the granules from the R4 reactor. For example, in two analyses carried out for one measurement area, the composition obtained was 19.3% P; 17.1% Mg; 2.2% Ca; 1.4% K for the first measuring point and 7.8% P; 2.3% Mg; 4.9% Ca; 8.9% K for the second.

### 2.3. Biological Properties of Biomass

**Figure 4:**
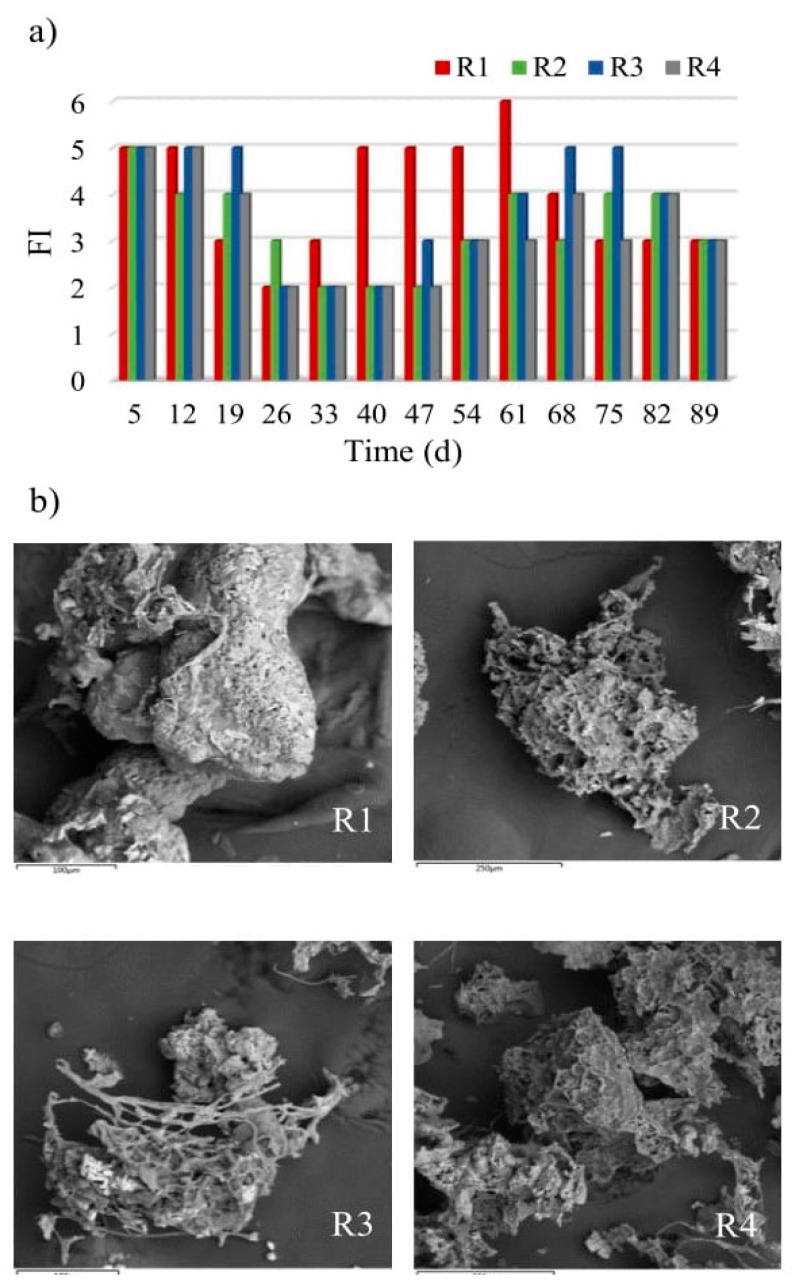
Changes in numbers of filamentous bacteria in reactors (**a**) and structure of granules resulting from their participation (**b**).

In the activated sludge serving as inoculum, there were free-flowing bacteria (sporadically), sedentary ciliates (*Vorticella campanula, Vorticella microstoma*), colonial ciliates (*Carchesium epistylis*), creeping ciliates (*Litonotus* sp., *Aspidisca costata*) and rotifers (*Philodina roseola, Rotifer vulgaris*). No filamentous bacteria were observed. The microscopic and microbiological studies showed differences between the reactors in terms of the number of filamentous bacteria that became visible from the 19th day of the study onwards. Throughout the experiment, lower numbers of filamentous microorganisms were observed in the reactors with powdered materials (Figure 4a).

The system was dominated by Type 021N bacteria, probably as a result of use glucose as a source of organic compounds in wastewater. Conducting research on sulfur bacteria strains, Williams and Unz [44] showed that Type 021N used glucose most intensively for growth. The presence of filamentous bacteria is recommended at the initial stage of biogranulation, as they provide the framework for granules [18]. However, their development en masse may lead to the formation of a specific form of biomass that is black in colour. Wang et al. [18] reported such observations, with rapid growth and accumulation of filamentous bacteria in a reactor found to ensure the dominance of the reactor by large black filamentous aggregates after just a few more days. In contrast, the application of powdered materials is seen to prevent the excessive washing out of the biomass from reactors, thus reducing the development of filamentous bacteria.

The SEM analysis of the cultured granules confirms the conclusion that the framework for granules is provided by filamentous bacteria. However, the differences in the extent to which this occurred could be noted. In the control reactor, where the number of filamentous bacteria was highest, these bacteria filled spaces between the framework of granules, as well as providing the framework itself, with consequences for the qualitative composition of the biocenosis in this reactor. Open spaces in the framework were present in the granules grown in the reactors with powdered materials, which allowed other microorganisms to co-exist and fill empty spaces inside the granules (Figure 4b).

From the 12th day of the study onwards, sedentary ciliates only represented a transient presence in the control reactor, an observation perhaps indicative of a failure to achieve stable operating conditions (for example too low sludge age, too high sludge organic load). In addition, from day 40 onwards, larger quantities of mastigophorans were present, perhaps as a reflection of the low sludge retention time (SRT), or even an overload with organic compounds. In addition, the low SRT in this reactor (3.7 ± 2.7 d) can be confirmed by the absence of testate rhizopods [45]. The reactors supplied with powdered materials did show higher biodiversity to the composition of biomass. A large number of creeping ciliates (mainly *Aspidisca and Litonotus* sp.), sedentary ciliates (mainly *Vorticella* sp.) and a few mastigophorans were observed, when it came to the protozoa. However, between days 61 and 75, the reactor with PG lacked sedentary ciliates, while testate rhizopods began to make their appearance in the R2, R3 and R4 reactors from day 47 on, their occurrence in turn being associated with an increased sludge retention time, as well as low F/M, good oxygenation and a low concentration of ammonium-nitrogen. In spite of a higher OLR being applied, rotifers were found to develop en masse from day 40 onwards in the R2 reactor and from day 33 in R3 and R4. It is typical to regard their presence as indicative of well functioning biomass. However, this phenomenon, accompanied as it was by periodic growth of nematodes, was not beneficial for biogranulation, given the way breakups of granules were to be observed in the R3 and R4 reactors, inter alia due to “prey” of these microorganisms. Observations indicate that powdered ceramsite ensured higher system stability.

The dehydrogenase activity (DHA) test in relation to biomass evidenced the differences between reactors not fully reflecting the microbiological biodiversity present in sludge. The highest average DHA value (of 71.05 µmol TF/g MLVSS) was recorded in the R1 reactor. For their part, Bernat et al. [46] reported that the dehydrogenase activity of the activated sludge was usually in the range 42–150 µmol TF/g MLVSS, ensuring a COD reduction efficiency of 85–90%. In the other reactors, the DHA values were of 32.01 (R2); 40.51 (R3) and 32.92 µmol TF/g MLVSS (R4). However, insufficient homogenisation of the biomass present most likely had its impact on the results. A correct action to obtain reliable DHA values would involve determination of the optimum trifenyltetrazolinum chloride (TTC) concentration in incubated samples, as well as incubation times for the biomass samples with increased numbers and sizes of granules. Miksch [47] presented this approach as a modification of the TTC test for activated sludge. It should be borne in mind that if TTC concentrations prove to be variable, this reflects the differences in qualitative composition, the structure of activated-sludge flocs and the physiological status of microorganisms.

**Figure 5:**
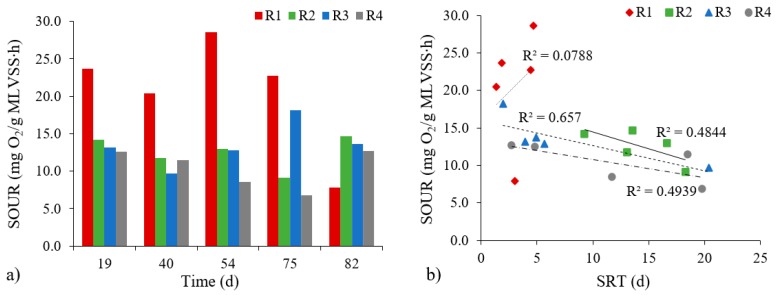
The rate of specific oxygen uptake by biomass in reactors (**a**) and changes between SOUR values depending on the SRT (**b**).

Evaluation of the physiological condition of biomass with the aid of the specific oxygen uptake rate (SOUR) test also showed differences (Figure 5a). The rate of specific oxygen uptake by biomass proved to be highest in the R1 reactor, and ranged from 20.42 to 28.57 mg O_2_/(g MLVSS∙h), albeit with a significant decrease to 7.85 mg O_2_/(g MLVSS∙h) on the 82nd day of the study. These results indicate that an insufficient biomass content occurred in the reactor in the final stage of the study. In addition, these results indicate that in the last days of research, there was an accumulation of the compounds which were toxic or inhibitory to certain groups of microorganisms. In the reactors with powdered materials, the SOUR values were at the levels 9.16–14.96 (R2); 9.67–18.16 (R3) and 6.81–12.65 mg O_2_/(g MLVSS∙h) (R4). Lower values for SOUR in these reactors mainly reflect higher biomass concentrations. Moreover, it was possible to note inverse correlations between the increase in SRT and the rate of specific oxygen uptake by microorganisms that were very high (in the R3 and R4 reactors) or high (R2) (Figure 5b). There was no correlation between the SOUR values and granule growth. The SOUR values in the research presented here for the reactors with powdered materials are, in fact, much lower than those obtained by He et al. [32] and Thanh et al. [31]. In the first case, the authors reported a rate of specific oxygen uptake by microorganisms in the reactor supported by yellow earth equal to 42.8 mg O_2_/(g MLVSS∙h). In the second case, where a bivalve shell carrier was used and OLR was 10.0 g COD/(L∙d), the rate of specific oxygen uptake by microorganisms was in the 47–58 mg O_2_/(g MLVSS∙h) range. Such large disparities in values may reflect the differences in the methodology adopted (not least the use of an additional source of organic compounds).

The content of calcium ions is a factor determining the aerobic bioactivity of granules, given the way these ions can induce dehydration at the surface of bacterial cells. This stimulates the aggregation of microorganisms and shortens the biogranulation time [42,48]. In the research presented here, the concentration of the calcium cation in raw wastewater was 0.27 mmol Ca/L. However, in the first operational cycle of the reactor, additional introductions to wastewater were of 0.127 mmol Ca/L and 0.045 mmol Mg/L from powdered ceramsite; 0.674 mmol Ca/L and 0.009 mmol Mg/L from powdered limestone, and 0.034 mmol Ca/L from powdered granite. The total amount of calcium cations, also in the initial days of the system in operation, was below 1 mmol Ca/L. The literature includes references to the effect that granules rich in Ca^2+^ are characterised by higher mechanical strength but are of a lower bioactivity [49]. The granules cultured in wastewater with 1 mmol Ca/L were characterised by a specific oxygen uptake rate of 9.55 ± 0.11 mg O_2_/(g MLVSS∙h). In contrast, the Ca^2+^ concentration equal to 0.5 mmol Ca/L was associated with a specific oxygen uptake rate equal to 22.26 ± 0.25 mg O_2_/(g MLVSS∙h) [49].

## 3. Materials and Methods

### 3.1. Reactor Set-up and Operation

**Figure 6:**
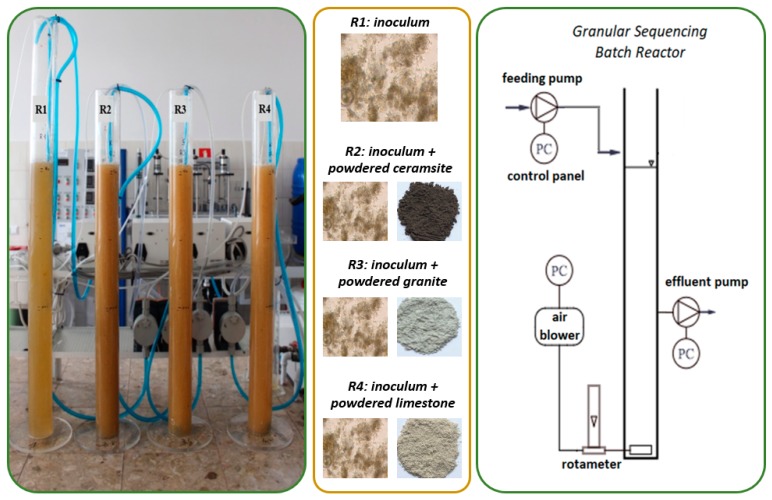
The test system.

Four identical GSBRs with the working volume of 3.0 L (internal diameter of 7.0 cm and effective height of 78.0 cm) were adopted for the research performed on a laboratory scale (Figure 6). The parameters of the experiment were adopted according to the studies of Czarnota et al. [20]. The average daily flow of wastewater through these was of 12.0 L/d at a volumetric exchange ratio equal to 0.5. The amount of wastewater fed during a cycle was 1.5 L, while the hydraulic retention time was 6 h. It should be noted that in R1, it was necessary to control the value of HRT, given the low biomass concentration. The R1 reactor was a control reactor, while others were supplied with the different powdered mineral materials at 3.0 g/L. Thus, powdered ceramsite was added to R2, powdered granite to R3 and powdered limestone to R4 (each material was added once at the reactor inoculation stage). The physicochemical properties of all the powdered mineral materials used are presented in Table 4.

The operational cycle of each reactor was 3 h, with 8 cycles per day. Each cycle included feeding (10 min), aeration (160 min), settling (4 min), decantation (5 min) and an idle phase (1 min). The air used in the reaction phase (aeration) was introduced through a fine-bubble air diffuser at the bottom of the reactor (flow 0.8 cm/s). The research was carried out for 89 days (with a 5-day initial adaptation period). The reactors were operated automatically through time-controllers.

The inoculum (accounting for 60% of the working volume of the reactor) was of used activated sludge from a nitrification tank of the WWTP in Rzeszów, Poland. MLSS and SVI of the seed sludge were of about 6.40 g MLSS/L and 135.2 mL/g, respectively. All reactors were fed with synthetic wastewater prepared in line with the chemical composition given by Thanh et al. [31]. The influent COD concentration of all reactors was 717.1 ± 62.6 mg O_2_/L at an OLR (organic loading rate) of 2.55 g COD/(L·d). The characteristics of the wastewater used in the research are presented in Table 5.

### 3.2. Analytical Method

MLSS (mixed liquor suspended solids), MLVSS (mixed liquor volatile suspended solids), SVI_5_ and SVI_30_ (the sludge volume index after 5 and 30 min of sedimentation) and SV (the sludge settling velocity) were measured using Standard Methods. The diameters of granules were determined using a method proposed by Arrojo [33], based on the use of *CellQ* image analysis software. The diameter was given by the mean value of the longest and shortest sections of a granule. Biomass density (as mass of granules per volume of granules) was determined using dextran blue, in line with the method described by Beun et al. [34]. However, given the way that the powdered mineral materials were being applied, measurements were made from day 33, and on selected dates. Computed tomography (Computer Tomograph Phoenix v | tome | x m by GE) was used to evaluate the differences relating to granule surface. Measurements were conducted for frozen granules insulated using a layer of styrofoam.

Determinations for extracellular polymeric substances (EPS) were performed using the sonication method [37,50]. The granule samples were homogenised with an ultrasonic disintegrator, centrifuged (with supernatant decanted), vortexed with 8.5% NaCl solution containing 0.22% formaldehyde, then centrifuged again with the supernatant again decanted. The liquid obtained was filtered through a 0.22 μm pore-diameter cellulose acetate filter. The EPS concentration in the filtrate was determined by the weight method. The granule composition was in turn analysed using an energy dispersion detector (EDS-type X-ray microanalysis system). Before that, the samples were lyophilised prior to being placed on microscopic tables covered with double-sided adhesive carbon tape.

The qualitative and quantitative composition of biomass microorganisms was characterised by way of microscopic observation (Olympus BX51 microscope), the relevant literature being used in the identification of organisms. The granular structure was determined by scanning electron microscopy (SEM) (MIRA Tescan microscope), with samples of biomass again being lyophilised prior to analysis. The activity of microorganisms in the biomass was evaluated by reference to biochemical measurements, including a determination of dehydrogenase activity (DHA) (µmol TF/g MLVSS), as well as the rate of specific oxygen uptake by biomass (SOUR) (mg O_2_/g MLVSS·h). The dehydrogenase activity in the biomass was determined by reference to the Polish PN-C-04616-8:2008 standard, entailing a spectrometric method with trifenyltetrazolinum chloride (a TTC test). Entailing a determination of how the concentration of dissolved oxygen in the aerated biomass taken from the reactor decreased, the SOUR test was performed in line with the methodology set out in relevant articles by Kristensen et al. [51] and Zielińska et al. [52]. An HACH flexi HQ30d multifunction meter was used to measure the dissolved-oxygen concentration.

### 3.3. Statistical Analysis

The STATISTICA 10 PL software was used in the statistical analysis. The statistical significance was determined based on Pearson’s linear correlation (relationships between pairs of variables were evaluated). The significance level assumed for the research was α = 0.05.

## 4. Conclusions

The results confirm the influence of applying powdered mineral materials on the selected physical, chemical and biological properties of granules. In the reactors to which the materials were added, the biomass sedimentation properties were more favourable, with the result being a more intensive process of granule formation. The largest and most stable granules were obtained in the reactor with powdered ceramsite, and their stability was confirmed by the fact that the density values were the highest noted. In turn, the reactors to which powdered granite and powdered limestone were supplied show problematic granule stability. In the initial stage of the study, powdered materials represent an additional “source” of hydrodynamic forces, inter alia influencing the production of EPS. In the reactors to which powdered ceramsite and limestone were added, the values for extracellular polymeric substances in the initial days of study were higher, probably as a result of Ca and Mg ions being present within the composition of these materials. The application of the powdered materials also curbed the excessive development of filamentous bacteria, mainly as a result of lower F/M values. The granules obtained were also found to differ both in terms of surface and structure. Fluffy surface with filamentous bacteria was characteristic for the granules cultured in the control reactor, while the reactors supplied with powdered materials produced granules with open spaces present in their framework. The SOUR values were more favorable in the reactors with materials than in the control reactor.

## Figures and Tables

**Table 1:** Sedimentation properties and biomass concentration in reactors.

Parameter	Units	R1	R2	R3	R4
MLVSS	(g MLVSS/L)	1.49 ± 0.36	4.12 ± 0.71	3.38 ± 1.93	3.94 ± 1.39
MLSS	(g MLSS/L)	2.28 ± 0.41	5.24 ± 0.78	4.46 ± 2.14	5.07 ± 1.54
SVI_5_	(mL/g)	156.27 ± 88.9	51.07 ± 22.9	81.71 ± 54.1	43.47 ± 29.3
SVI_30_	(mL/g)	96.6 ± 38.2	39.8 ± 8.6	55.5 ± 27.4	32.8 ± 10.7
SVI_5_/SVI_30_	(-)	1.6 ± 0.3	1.3 ± 0.3	1.4 ± 0.4	1.3 ± 0.4
SV	(m/h)	4.0 ± 0.4	15.4 ± 6.1	10.7 ± 2.9	13.1 ± 4.4

**Table 2:** Comparison of biomass in reactors on the 89th day of the experiment.

Parameter	Units	R1	R2	R3	R4
Average diameter	(µm)	200.2	783.1	399.0	430.0
MLVSS	(g MLVSS/L)	1.64	3.34	1.27	1.55
MLSS	(g MLSS/L)	2.39	4.23	1.98	2.30
Density of granules	(g MLVSS/L_granules_)	14.61	37.26	16.69	21.45
Number of granules*	(-)	8∙10^7^	1.1∙10^6^	6.9∙10^6^	5.2∙10^6^
Specific surface area of the granules*	(m^2^/m^3^)	3369**	687	1145***	1010***

* the parameters were determined according to the methodology presented by Beun et al. [34]. ** no full biogranulation of the biomass. *** a reduction in the diameter of the granules was observed.

**Table 3:** Map of the distribution of selected chemical elements in the area of granules.

Reactor	Element		
	Calcium	Phosphorus	Magnesium
R1	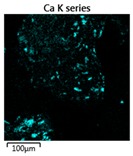	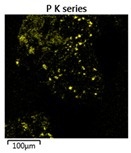	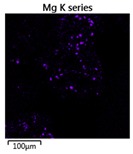
R2	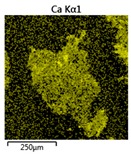	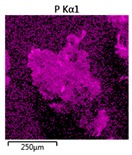	
R3	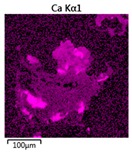	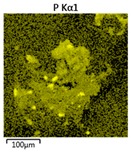	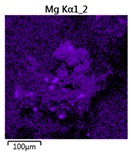
R4	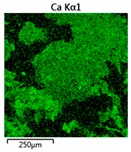	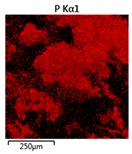	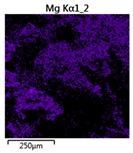

**Table 4:** Characteristics of powdered mineral materials.

Parameter, Units	Powdered Granite (PG)	Powdered Ceramsite (PK)	Powdered Limestone (PL)
Sphericity	low sphericity	low sphericity	high sphericity
Diameter: d_10_; d_50_; d_90_, µm	3.194; 26.817; 100.470	3.643; 24.110; 85.279	1.865; 33.915; 189.720
Apparent density, g/cm^3^	2.6197	2.6182	2.1949
Specific surface area, m^2^/g	1.792	5.183	1.760
Chemical composition, mg/g	Ca: 13.89; Mg: 1.46; Fe: 10.28; Si: 326.28	Ca: 75.90; Mg: 21.61; Fe: 45.15; Si: 216.30	Ca: 691.04; Mg: 4.61; Fe: 1.68; Si: 5.58
Substance leaching, µg/g	Ca: 50.34; Mg: 2.38; Fe: 1.25; Si: 50.98	Ca: 451.65; Mg: 97.87; Fe: 0.50; Si: 23.01	Ca: 87.75; Mg: 12.67; Fe: 0.21; Si: 7.00
Settling velocity, m/h	approx. 12.0	approx. 9.0	approx. 12.0

**Table 5:** Characteristics of synthetic wastewater.

Parameter	Units	Component	Average	Standard Deviation	Minimum	Maximum
COD	mg O_2_/L	glucose	717.1	62.6	615.0	785.0
TP	mg P/L	KH_2_PO_4_	9.73	1.05	8.55	11.6
TN	mg N/L	NH_4_Cl	44.0	3.0	40.3	50.3
COD/TP ratio	-	-	74.9	12.8	53.8	89.7
COD/TN ratio	-	-	16.3	1.6	13.8	18.7
